# CpG Methylation across the adipogenic *PPARγ* gene and its relationship with birthweight and child BMI at 9 years

**DOI:** 10.1186/s12881-016-0365-4

**Published:** 2017-01-26

**Authors:** Vitaly Volberg, Paul Yousefi, Karen Huen, Kim Harley, Brenda Eskenazi, Nina Holland

**Affiliations:** Center for Environmental Research and Children’s Health (CERCH), School of Public Health, University of California, 733 University Hall, Berkeley, CA 94720-7360 USA

**Keywords:** Epigenetics, Birth cohort study, *PPARγ*, Obesity, Newborns, Children, Whole blood, DNA methylation

## Abstract

**Background:**

To examine methylation of the peroxisome proliferator-activated receptor γ (*PPARγ*) gene and its relationship with child weight status, at birth and 9 years.

**Methods:**

We measured *PPARγ* methylation across 23 CpG sites using the Infinium Illumina 450 k array for children from the Center for the Health Assessment of Mothers and Children of Salinas (CHAMACOS) cohort at birth (*N* = 373) and 9 years (*N* = 245).

**Results:**

Methylation level correlation patterns across the 23 *PPARγ* CpG sites were conserved between birth and 9-year ages. We found high inter-CpG correlations between sites 1–3 (methylation block 1) and also between sites 18–23 (methylation block 2) for both time points, although these patterns were less pronounced at 9 years. Additionally, sites 1–3 (north shore) had the highest intra-CpG correlations over time (*r* = 0.24, 0.42, and 0.3; *P* = 0.002, *P* < 0.001, *P* < 0.001, respectively). *PPARγ* methylation levels tended to increase with age, and the largest differences were observed for north shore sites (7.4%). Adjusting for sex, both site 1 and site 20 (gene body) methylation at birth was significantly and inversely associated with birth weight (β = −0.13, *P* = 0.033; β = −0.09, *P* = 0.025, respectively). Similarly, we found that site 1 and site 20 methylation at 9 years was significantly and inversely associated with 9-year BMI z-score (β = −0.41, *P* = 0.015; β = −0.23, *P* = 0.045, respectively).

**Conclusion:**

Our results indicate that *PPARγ* methylation is highly organized and conserved over time, and highlight the potential functional importance of north shore sites, adding to a better understanding of regional human methylome patterns. Overall, our results suggest that *PPARγ* methylation may be associated with child body size.

**Electronic supplementary material:**

The online version of this article (doi:10.1186/s12881-016-0365-4) contains supplementary material, which is available to authorized users.

## Background

It has been hypothesized that prenatal environmental exposures may cause lasting epigenetic changes during child development, leading to adverse health outcomes in later life [1, 2]. Epigenetics refers to heritable changes regulating gene expression that do not affect the DNA base pair sequence. DNA methylation is the most commonly studied epigenetic mark [3–6]. This modification involves attachment of a methyl group to the cytosine base within cytosine-guanine dinucleotides, also known as ‘CpG methylation’. Higher CpG methylation (hypermethylation) within the promoter region of a gene can reduce gene expression [7].

Our understanding of how the human methylome is organized is rapidly evolving and current literature outlines a classification system for CpG sites, highlighting that their function may be intimately related to their location in the gene [8, 9]. For example, approximately 70% of gene promoter regions are thought to contain a CpG island – a region with densely concentrated CpGs that typically have low levels of methylation [10]. CpG sites flanking the island are located in regions termed north shore (upstream, 5′ end) and south shore (downstream, 3′ end) and are thought to be particularly important in regulating gene expression [6, 9].

There is a growing interest in examining interactions between environmental and genetic factors on differential CpG methylation. A classic example is from animal studies on the *Agouti* mouse, where hypomethylation of the intracisternal A particle (IAP) increases expression of the *Agouti* gene, resulting in yellow coat color and obese phenotype. Using this model, Waterland et al. [11] showed that inheritance of the *Agouti* gene was associated with trans-generational amplification of obesity and that maternal methyl-donor supplementation could prevent this effect.

In humans, DNA methylation has been proposed to mediate direct intra-uterine associations between maternal and offspring phenotypes. Differential DNA methylation has been reported when assessing offspring exposed *in utero* to extreme maternal undernutrition [12, 13], maternal morbid obesity [14] and less extreme maternal underweight and maternal obesity [15]. However, several important challenges remain. There is an ongoing effort to determine the causal direction between DNA methylation and an offspring phenotype. In their 2016 study, Richmond et al. apply a causal framework to parse out whether *HIF3A* methylation has a causal effect on BMI or vice versa [16]. Their results argue for the potential of a phenotype to affect methylation status and highlight the potential for inter-generational influence of maternal BMI on offspring methylation, possibly confounding the offspring *HIF3A* methylation and obesity association.

Another important challenge has been replication of results from epigenome-wide association studies (EWAS) studies. For example, the EWAS study by Sharp et al. identified 28 CpGs in newborns that were associated with maternal pre-pregnancy BMI. Four of these hits had previously been reported in literature, but their results did not replicate the direction and magnitude of the earlier analyses [17]. Additionally, in their EWAS, Aslibekyan et al. found only 8 CpGs in 3 genes (*CPT1A*, *PHGDH*, *CD38*) associated with body mass index (BMI) in adults that withstood replication and multiple testing adjustment [18].

To avoid the limitations of multiple testing, candidate genes can be selected *a priori*. With respect to obesity development, the peroxisome proliferator-activated receptor γ *(PPARγ*) gene may play a critical role, functioning as the only gene that is both necessary and sufficient for fat cell production [19, 20]. *PPARγ* upregulation has been linked to improvement of critical metabolism-related hormones (increased adiponectin and decreased leptin) and increased insulin sensitivity at the expense of greater body weight in adults [21] and animals [22–24]. Importantly, while methylation affects *PPARγ* expression in animal and *in vitro* studies, only limited human data on *PPARγ* methylation, its relationship with obesity and/or with perinatal factors are available [25, 26].

In the Center for the Health Assessment of Mothers and Children of Salinas (CHAMACOS) cohort, we have previously examined a subset of *PPARγ* CpG sites and their relationship with gene expression in a cohort of children with a high prevalence of obesity [27]. We reported that hypomethylation of the *PPARγ* CpG site cg10499651 was associated with increased *PPARγ* expression as measured by both real-time polymerase chain reaction (RT-PCR) and nCounter assays. In the current investigation, we build on this finding and add to current data gaps on *PPARγ* methylation and its relationship with obesity. We use the Illumina 450 k assay to examine methylation of 23 CpG sites spanning the *PPARγ* promoter and gene body regions in children at birth (*N* = 373) and at 9 years (*N* = 245) and 1) analyze the correlation structure between the 23 *PPARγ* CpG sites, 2) characterize associations between perinatal factors, including maternal pre-pregnancy BMI, and *PPARγ* methylation at birth and at 9 years, and 3) examine associations between *PPARγ* methylation, child birthweight and BMI at 9 years.

## Methods

### Subject and study design

The CHAMACOS study is a longitudinal birth cohort designed to assess the health effects of pesticides and other environmental exposures on growth and development of primarily Mexican-American children living in Salinas Valley, an agricultural region of California [28, 29]. Mothers were enrolled during pregnancy between October 1999 and October 2000, with 537 mother-child pairs in the study at delivery and 327 pairs participating at the 9-year visit. Eligible women were ≥18 years of age, <20 weeks gestation at enrollment, English or Spanish speaking, eligible for low-income health insurance (Medi-Cal) and planning to deliver at the county hospital. Women were interviewed twice during pregnancy, shortly after delivery, and when their children were 6 months, and 1, 2, 3½, 5, 7, and 9 years of age. This study used a subset of the CHAMACOS cohort (373 children at birth and 245 children at 9 years) who had blood samples available for methylation analyses. Study protocols (2010-01-620 & 2010-03-949) ethics were approved by the University of California, Berkeley Committee for Protection of Human Subjects. Written informed consent was obtained from all mothers and assent was provided by the children at the 9-year assessment.

### Questionnaire data

Interviews were conducted in Spanish or English by bilingual, bicultural trained interviewers. Maternal age was assessed during the first prenatal interview at 14 ± 5 weeks gestation. Maternal pre-pregnancy BMI was calculated using the mother’s self-reported pre-pregnancy weight and measured height. Data on infant birth weight and gestational age were obtained from delivery medical records abstracted by a registered nurse.

### Anthropometric measurements

An electronic scale (Tanita Mother-Baby Scale Model 1582, Tanita Corp.) was used to measure child weight at the 9-year visit. Child 9-year height was measured in triplicate using a stadiometer (Seca 222) and the average of measurements was used. Child height and weight were converted to age- and sex-specific BMI z-scores using the 2000 Centers for Disease Control and Prevention (CDC) child growth data and children were categorized as normal weight, overweight, or obese using the sex and age-specific cutoffs (85th and 95th percentile, respectively) [30]. Monthly rate of weight gain during the first 6 months of life was calculated as weight at the 6-month visit minus birth weight divided by exact age in months at the 6-month visit and reported in 100 grams/month. This approach to examining infancy weight gain has been previously validated [31].

### Child CpG methylation measurement

DNA was isolated from blood clots previously collected and stored at −80 °C using the QIAamp DNA blood maxi kit (Qiagen, CA). To measure CpG methylation, we used the Infinium Illumina 450 k array, which is based on multiplexed genotyping of bisulfite converted genomic DNA. This technology is currently considered the leading method to measure genome-wide methylation, providing both broad and dense coverage, in total interrogating 485,577 CpG sites over 99% of RefSeq genes. The workflow involves bisulfite conversion of DNA, performed using Zymo Bisulfite Conversion Kits (Zymo Research, Orange, CA). Subsequently, each sample is whole-genome amplified, enzymatically fragmented, purified and applied to the BeadChips according to the Illumina methylation protocol. BeadChips were processed with robotics and analyzed using the Illumina Hi-Scan system at the Genomics Core. Samples included in the analysis had detection *P* values below 0.01 for 95% of CpG sites and poor performing CpG sites with *P* value > 0.01 were excluded. Raw signal intensities were background corrected and then normalized for color-channel bias using the all sample mean normalization method as described by Yousefi et al. (2013) [32]. Beta mixture quantile normalization was also applied to make interpretation between type I and type II probes comparable [33]. One of the CpG sites in our analysis (CpG site 13, cg04632671) was found to have a common single nucleotide polymorphism (SNP), minor allele frequency > 5%, within 50 base pairs in the Mexican ancestry in Los Angeles, California, (MXL) HapMap population. In our sensitivity analysis, excluding this site had no impact on our results and a decision was made to retain this site. Additionally, although we did not have data on the rs1801282 Pro12Ala *PPARγ* SNP, adjusting our analyses for potential effects of admixture did not affect the relationships between *PPARγ* DNA methylation and birthweight or BMI in our study.

### Cell composition

To examine the relationship of blood cell composition with CpG methylation in *PPARγ* CpG sites, we performed differential cell counts in a subset of cord samples (*N* = 111) as described previously [34]. To prepare heparinized whole blood smears, we used the “gold standard” Wright-Push blood smearing technique followed by staining utilizing a DiffQuikVR staining kit [35]. At least 100 cells were scored for each slide, and a percentage of each cell type (lymphocytes, monocytes, neutrophils, eosinophils, and basophils) was used for data analysis. The coefficient of variation (CV) for the repeat scoring in this subset was less than 10%.

Whole blood smears were not available for differential cell count in 9-year-old CHAMACOS children. For these children we used the Bioconductor R package minfi (v1.10.2) to estimate the distribution of six different white blood cell types (CD8+ T and CD4+ T lymphocytes, CD56+ natural killer cells, CD19+ B cells, CD14+ monocytes, and granulocytes) based on their methylation signatures in 450 k data [36]. We did not use minfi to estimate cell composition at birth as we have previously shown that proportions of white blood cells in newborns are significantly different from the adult reference samples on which minfi estimates are based [34, 37, 38].

For comparison of cell type composition in cord bloods to those estimated by minfi in 9-year-olds, we used proportions of lymphocytes, granulocytes, and monocytes. For minfi estimates, this required summation of the frequencies for CD8+ T, CD4+ T, natural killer cells, and B cells to calculate the proportion of lymphocytes. For differential cell count, proportions of neutrophils, eosinophils, and basophils were summed to give an estimate of granulocytes.

Additionally, we used data from four separate cohorts, including Bakulski et al. 2016 [39], characterizing cell composition in cord blood in relation to DNA methylation [40–42]. Re-running our analysis using data from these four cohorts as a reference did not change our findings.

### Statistical analyses

Our statistical analyses used methylation levels expressed as M-values, which are calculated as the log_2_ ratio of the intensities of methylated probe to unmethylated probe, M-value = log_2_(beta/(1-beta)) [43]. In addition, we also presented relative methylation betas (% methylation) in some of the tables for ease of interpretation. Importantly, these untransformed values were not used in analyses given the reported heteroscedasticity for highly methylated or unmethylated CpG sites [43]. To analyze *PPARγ* methylation structure across the 23 sites, we plotted methylation levels by site and examined inter-site correlations at birth and 9 years using Pearson’s correlation coefficients (r). Linkage disequilibrium (LD) methylation blocks were established based on several criteria slightly modified from Shoemaker et al. (2010) and Liu et al. (2014): (i) they had to contain at least 3 contiguous CpG sites and (ii) at least 50% of the CpG site pairs had to have methylation levels that were highly correlated with each other (r^2^ > 0.4) [44, 45]. Additionally, we calculated correlation coefficients for each CpG site comparing values at birth to 9 years and tested whether *PPARγ* methylation levels changed from birth to 9 years using generalized estimating equations (GEE) and whether methylation levels differed at each site and at each age by sex using Student’s *t*-Test. We examined associations between *PPARγ* methylation at birth and child birthweight and *PPARγ* methylation at 9 years and 9-year BMI z-score for each of the 23 CpG sites. We used directed acyclic graphs (DAGs) to select our covariates for multiple regression models. We examined whether methylation levels varied by gestational age, maternal age, maternal BMI, parity, and weight gain in the first six months of life [46–50]. Bonferroni correction was used to account for multiple testing. Testing for associations between 23 CpG sites with 4 variables at birth and 5 variables at the 9-year time point comprises 207 tests. Thus for this analysis, we used an adjusted alpha of 0.05/207 = 2.4E-4. Statistical analyses were conducted using STATA 12 (College Station, TX) for Windows and R statistical software (R Foundation for Statistical Computing, Vienna, Austria).

## Results

### Maternal and child characteristics

Our study sample included a total of 444 children who had DNA samples available for methylation analysis at birth and/or age 9 in addition to birthweight and BMI at 9 years. Of these children, 174 had samples available at both time points, 199 had samples only at birth, and 71 had samples at age 9 only. Overall, there were 373 children available for analyses at birth and 245 children at 9 years.

At pregnancy, mothers tended to be young (25.6 years, 95% CI 25.1, 26.1 years) and overweight or obese (61%), with an average BMI of 27.0 kg/m^2^ (95% CI of 26.5, 27.5 kg/m^2^). Of the 444 children in this study, there were similar numbers of boys (*N* = 221) and girls (*N* = 223) (Table 1). Mean gestational duration was 38.9 weeks (95% CI 38.7, 39.0 weeks) and mean birthweight was 3.46 kg (95% CI 3.41, 3.5 kg). Their average weight gain in the first 6 months of life was 0.73 kg (95% CI 0.71, 0.75 kg). At 9 years, the majority of children were overweight or obese (56%), with mean BMI of 20.7 kg/m^2^ (95% CI of 20.1, 21.3 kg/m^2^) and mean BMI-z-score of 1.11 (95% CI of 0.98, 1.24). We did not find any statistically significant differences between the maternal and child characteristics, including anthropometric measures, in this subset compared to the overall CHAMACOS dataset.

**Table 1:** CHAMACOS maternal and child characteristics

Cohort Characteristics	Number	Mean	Range	95% CI
Sex (male/female)	221/223	---	---	---
Gestational age (weeks)	444	38.9	33–42	38.7, 39.0
Parity	444	1.3	0–9	1.1, 1.4
Birth weight (kg)	444	3.46	1.93–4.89	3.41, 3.50
Weight gain in 1^st^ 6 months (kg)	229	0.73	0.34–1.22	0.71, 0.75
BMI at 9 years (kg/m^2^)	240	20.7	13.9**–**33.8	20.1, 21.3
BMI z-score	240	1.11	−1.57–2.70	0.98, 1.24
Maternal age at pregnancy (years)	444	25.6	18**–**43	25.1, 26.1
Maternal pre-pregnancy BMI (kg/m^2^)	444	27.0	17.7**–**57.3	26.5, 27.5

*BMI* body mass index

### *PPARγ* methylation

Additional file 1: Figure S1 shows the distribution of 23 CpG sites (red squares) measured by the Illumina Methylation 450 k array across the *PPARγ* gene promoter and body. Blue squares indicate all other CpG sites (*N* = 183). This figure was generated using the DNA sequence provided in the Genome Reference Consortium Human Build 38 patch release 2 (GRCh38.p2) and serves as a visual reference showing the relative locations of the 23 CpG sites. Illumina annotation classifies sites 1–3 as north shore (cg01412654, cg25929976, and cg18063278), 4–15 as island (cg26364899, cg27095527, cg06573644, cg21946299, cg23514324, cg04748988, cg15722404, cg13518792, cg09405169, cg04632671, cg07556134, and cg18887186), 16–17 as south shore (cg21859053 and cg04908300), 18 as south shelf (cg16827534), 19 as 5′ untranslated region (5′ UTR) (cg16197186), and 20–23 as *PPARγ* gene body (cg18537222, cg07895576, cg07676920, and cg10499651) [8, 9].

**Fig. 1:**
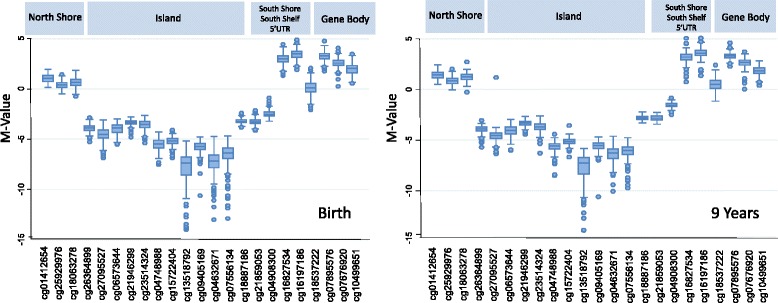
Methylation M-values across 23 *PPARγ* CpG Sites at birth and 9-years. Sites are labeled according to their Illumina 450K annotation. Methylation levels are highly conserved comparing the two time points

Figure 1 shows methylation M-Values across the 23 *PPARγ* CpG sites at birth and 9-years. As expected, CpG sites in the north shore (average beta 61.4% at birth, 68.8% at 9 years), south shore (average beta 12.5% at birth, 18.8% at 9 years) and shelf (average beta 88.2% at birth, 89.5% at 9 years), 5′UTR (average beta 91.2% at birth, 91.9% at 9 years) and gene body (average beta 76.5% at birth, 78.0% at 9 years) tended to be highly methylated while island sites had significantly lower methylation (average beta 4.5% at birth, 4.6% at 9 years). Interestingly, we observed a highly conserved pattern of methylation across the 23 sites comparing both time points. For example, sites 21, 22, 23 follow the same pattern of decreasing methylation at birth (90.2%, 85.1%, and 78.9%, respectively) and at 9 years (90.7%, 85.7%, and 77.7%, respectively).

### *PPARγ* methylation blocks

**Fig. 2:**
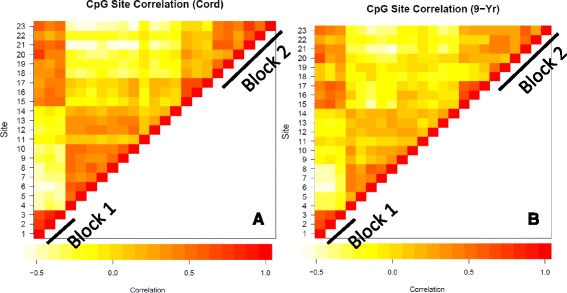
Correlations for the 23 *PPARγ* CpG sites spanning the promoter and gene body region at (**a**) birth and (**b**) 9 years. White/yellow squares represent none/poor correlations, while orange/red squares represent moderate/high correlations. Two methylation blocks were identified for both time points, Block 1 consists of north shore sites 1-3 and Block 2 consists of south shelf, 5’ untranslated region (5’UTR), and gene body sites 18-23

There is a growing body of literature showing that neighboring CpG sites are often highly correlated with each other [45, 51]. We examined inter-CpG correlations for the 23 *PPARγ* sites and our results are presented in Fig. 2. Both at birth and 9-years, we identified two methylation blocks. Block 1 was comprised of north shore sites 1–3 spanning approximately 1 kilobase (kb) pairs. Block 2 contained sites 18–23 located in the south shelf, 5′UTR, and gene body, and spanned approximately 130 kb. Additionally, we noted that correlations within the blocks were consistently stronger at birth compared to 9 years. At birth, correlations ranged from 0.66 to 0.79 and from 0.21 to 0.79 for blocks 1 and 2, respectively, while at 9 years, block 1 ranged from 0.55 to 0.69 and block 2 ranged 0.12 to 0.57. Interestingly, we also found that CpG site 1 was highly correlated with methylation at gene body sites 20–23 at both birth (*r* = 0.74, 0.54, 0.31, and 0.59, all *P* < 0.001) and 9-years (*r* = 0.66, 0.50, 0.30, and 0.53, all *P* < 0.001).

### *PPARγ* methylation by age

**Fig. 3:**
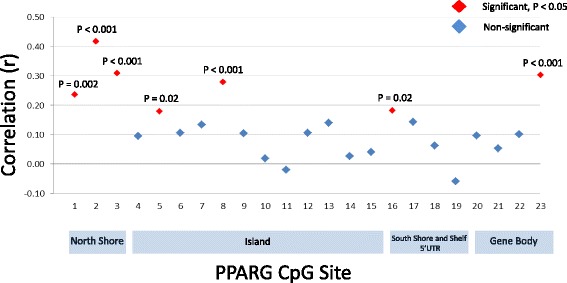
Correlations between birth and 9-year methylation values for the 23 *PPARγ* CpG Sites. All three north shore sites were significantly correlated over time

Figure 3 shows correlations for *PPARγ* CpG sites between the birth and 9-year time points. We found significant correlations for all three of the north shore sites (*r* = 0.24, 0.42, and 0.31; *P* = 0.002, <0.001, and < 0.001, respectively). Island CpG sites 5 and 8 (*r* = 0.18 and 0.28; *P* = 0.02 and <0.001, respectively), south shore site 16 (*r* = 0.18; *P* = 0.02), and gene body site 23 (*r* = 0.3; *P* < 0.001) were also significantly correlated over time. We used GEE to account for repeated measures over time and found that for all regions (north shore, island, south shore, south shelf, 5′UTR and gene body) methylation values had statistically significant increases with age. The greatest increases were observed for north and south shore sites, 7.4% and 6.3%, respectively. Averaging over all 23 sites, methylation levels had a statistically significant increase of 2% over the 9-year span. Increases in methylation remained statistically significant for all regions adjusting for cell composition (data not shown).

### *PPARγ* methylation by sex

Table 2 shows methylation betas for all 23 *PPARγ* CpG sites stratified by sex at both the birth and 9-year time points. For all three north shore sites, females had significantly higher methylation betas compared to males at both birth (site 1: 67.8 vs. 65.8, *P* = 0.002, site 2: 58.1 vs. 55.6, *P* < 0.001, site 3: 62.1 vs. 59.1, *P* < 0.001) and 9 years (site 1: 73.1 vs. 71.5, *P* = 0.018, site 2: 65.4 vs. 62.5, *P* < 0.001, site 3: 71.1 vs. 68.3, *P* < 0.001). Females also had significantly higher methylation at 9 years at site 17 (25.7 vs. 24.8, *P* = 0.03) and at birth at site 18 (88.6 vs. 87.8, *P* = 0.021).

**Table 2:** PP*PPARγ* methylation by sex

*PPARγ* Site	CpG Illumina ID	Classification^a^	Methylation at birth (%)			Methylation at 9 years (%)		
			Male (*N* = 188)	Female (*N* = 185)	*P* value^b^	Male (*N* = 113)	Female (*N* = 132)	*P* value^b^
1	cg01412654	North Shore	65.8	67.8	0.002	71.5	73.1	0.018
2	cg25929976	North Shore	55.6	58.1	<0.001	62.5	65.4	<0.001
3	cg18063278	North Shore	59.1	62.1	<0.001	68.3	71.1	<0.001
4	cg26364899	Island	6.3	6.3	0.931	6.0	6.2	0.156
5	cg27095527	Island	4.3	4.5	0.289	4.0	4.6	0.380
6	cg06573644	Island	6.2	6.4	0.434	5.8	5.8	0.887
7	cg21946299	Island	9.0	8.9	0.445	9.1	9.0	0.724
8	cg23514324	Island	8.0	8.0	0.943	7.4	7.3	0.626
9	cg04748988	Island	2.3	2.3	0.636	2.0	2.0	0.570
10	cg15722404	Island	2.8	2.7	0.430	2.8	2.7	0.119
11	cg13518792	Island	1.0	1.0	0.297	1.0	1.0	0.564
12	cg09405169	Island	1.9	1.8	0.550	2.1	2.1	0.707
13	cg04632671	Island	1.0	1.1	0.115	1.4	1.2	0.054
14	cg07556134	Island	1.3	1.3	0.797	1.5	1.5	0.457
15	cg18887186	Island	9.8	9.9	0.372	12.5	12.5	0.709
16	cg21859053	South Shore	9.4	9.5	0.459	12.3	12.4	0.657
17	cg04908300	South Shore	15.4	15.9	0.107	24.8	25.7	0.030
18	cgl6827534	South Shelf	87.8	88.6	0.021	89.7	89.4	0.506
19	cg16197186	5′ UTR	91.1	91.3	0.398	92.0	91.9	0.996
20	cg18537222	Gene Body	51.5	52.1	0.612	57.1	58.4	0.345
21	cg07895576	Gene Body	90.1	90.3	0.244	90.7	90.6	0.707
22	cg07676920	Gene Body	85.1	85.0	0.930	85.9	85.4	0.513
23	cg10499651	Gene Body	79.0	78.8	0.632	77.1	78.2	0.084

*PPARγ* peroxisome proliferator-activated receptor gamma

*UTR* untranslated region

^a^Based on Illumina 450 K annotation

^b^Student’s *t*-Test

### Associations between perinatal factors and *PPARγ* methylation

Additional file 2: Table S1 and Additional file 3: Table S2 show regression results for testing associations between the selected perinatal characteristics and *PPARγ* methylation at birth and 9 years, respectively. Of note, at both birth and 9 years, methylation at *PPARγ* site 22 was inversely associated with maternal pre-pregnancy BMI. However, this relationship was not significant after adjustment for multiple testing. All other perinatal variables were not associated with methylation at any of the sites.

### Relationship between *PPARγ* methylation and birth weight and 9-year BMI z-score

Table 3 summarizes our analysis of the relationships between *PPARγ* methylation and child birthweight and BMI at age 9. Given evidence in literature highlighting importance of island-flanking CpG sites, our analyses were focused on sites 1–3 and 16–23. Adjusting for sex, we found that methylation at birth for both site 1 and site 20 was significantly and inversely associated with birth weight (β = −0.13, *P* = 0.033; β = −0.09, *P* = 0.025, respectively). Similarly, we found methylation at 9 years at site 1 and site 20 was significantly and inversely associated with 9-year BMI z-score (β = −0.41, *P* = 0.015; β = −0.23, *P* = 0.045, respectively). We subsequently tested whether methylation at birth at either of these sites was associated with 9-year BMI z-score but did not find any significant associations. Adjusting for cell composition did not appreciably change results of these analyses.

**Table 3:** Associations between *PPARγ* methylation and child size at birth and 9 years of age

CpG Site Birth	Birth Weight (kg) (*N* = 373)		9-Year BMI i-Score (*N* = 240)	
	Beta^a^	*P* value	Beta	*P* value
1	−0.13	0.033	−0.41	0.015
2	−0.06	0.367	0.11	0.514
3	0.01	0.825	−0.08	0.625
16	−0.11	0.191	0.43	0.099
17	−0.04	0.537	0.40	0.151
18	−0.02	0.739	−0.05	0.689
19	−0.05	0.400	−0.16	0.260
20	−0.09	0.025	−0.23	0.045
21	−0.01	0.917	−0.05	0.825
22	0.04	0.517	−0.17	0.293
23	−0.01	0.987	−0.14	0.380

*PPARγ* peroxisome proliferator-activated receptor gamma

*BMI* body mass index

^a^Adjusted for sex

## Discussion

In this study, we aimed to address several knowledge gaps on the 1) correlation structure of *PPARγ* methylation, 2) relationships between perinatal factors and *PPARγ* methylation, and 3) associations between *PPARγ* methylation, birth weight and child BMI. We found that *PPARγ* methylation displays a highly conserved pattern and report on two methylation blocks comprised of sites 1–3 (block 1) and 18–23 (block 2) present at both birth and 9-year time points. Additionally, we observed high intra-CpG correlations comparing the birth to 9-year time points for all three north shore CpG sites. With respect to aim 2, we found that none of the perinatal variables examined, including gestational age, parity, maternal age and pre-pregnancy BMI and in addition, for 9 years, weight gain in the first 6 months, were significantly associated with *PPARγ* methylation at either birth or 9 years. Further, we observed that girls had significantly greater methylation at north shore sites 1–3 compared to boys at both time points. Adjusting for sex, we found that methylation at birth for sites 1 and 20 was significantly and inversely associated with birth weight. Similarly, we found that methylation at these sites at 9 years was also significantly and inversely associated with 9-year BMI z-score. Taken together, these results indicate that *PPARγ* methylation may be involved in regulating child body size and highlight the potential functional importance of north shore sites.

*PPARγ* CpG organization is typical of many other genes, with its promoter region containing a CpG island flanked by north and south shore sites [52]. Additionally, in agreement with studies showing complex inter-CpG correlations over both short and long regions, *PPARγ* contained two methylation blocks spanning 1 kb over the north shore (block 1) and 130 kb over the south shore, 5′ UTR, and gene body (block 2) [45, 53]. Interestingly, we also found that north shore CpG site 1 from block 1 was correlated with methylation at sites 20–23 from block 2. There is a growing understanding that the location of a particular CpG site may be functionally important and several studies have highlighted the role of shore sites in gene expression, tissue differentiation, and overall phenotype [9, 54, 55]. For example, Doi et al. (2009) showed that CpG shore methylation distinguished between several cell lines, including brain, liver, spleen cells, their pluripotent stem cells and parental fibroblasts [54]. Similarly, Irizarry et al. (2009) showed that most methylation changes associated with colon cancer occurred in CpG shores [9]. Our observations of methylation blocks surrounding the *PPARγ* CpG island and high correlations between the north shore and gene body sites add evidence that shore sites may be of particular relevance in regulating biological pathways.

With respect to changes in CpG methylation over time, although some reports indicate stable methylation patterns [53, 56] others do not [57–59]. In their analyses of blood samples from the Netherlands Twin Register, Talens et al. (2010) showed that of 8 regions examined, 5 displayed stable methylation patterns for up to 20 years [56]. Additionally, using Illumina 450 k data, we have previously shown that methylation across 16 paraoxonase 1 gene (*PON1*) shore, shelf, and island sites was highly conserved comparing birth and 9-year time points [53]. On the other hand, Fraga et al. (2004) showed that while 3-year-old monozygotic (MZ) twins showed relatively few epigenetic differences, there was considerably larger variability in older twin pairs [57]. Our results indicate that *PPARγ* methylation is stable over the birth to 9-year period and that even minute differences between CpG sites are conserved.

Although the pattern of CpG sites remained similar over time (Fig. 1), we found that north shore sites had slightly but significantly higher beta values (7.4%) at 9 years compared to birth. Previous literature has identified both hypo and hyper-methylation changes with age and taken together, these findings suggest that different genomic regions may have varying stability over time [5, 28, 60, 61]. Additionally, we observed small differences by sex, with girls having slightly higher methylation compared to boys, at both birth and 9-year time points. However, these differences were limited to north shore sites 1–3. Although the significance of this remains unclear, our previous work using 450 k data identified that overall ~ 3% of CpG sites are differentially methylated by sex and are enriched for genes related to nervous system development and behavior [53]. Interestingly, Hall et al. (2014) showed that genome-wide CpG methylation in pancreatic islets differentially clustered between males and females, suggesting that methylation may be involved in sex-specific metabolic differences [62]. Our results are in line with this and overall show that *PPARγ* CpG methylation is carefully maintained, emphasizing its potentially important role in regulating *PPARγ* function.

In addition to data gaps on methylation structure and organization, very little is known about the epigenetic changes that accompany obesity development. Our report of an inverse relationship between *PPARγ* methylation and body size is consistent with the idea that higher methylation downregulates *PPARγ,* suppressing adipogenesis. To date, few studies have examined these relationships in *PPARγ*, providing mixed results [63, 64]. Yan et al. (2014) examined *PPARγ* gene expression and methylation in offspring of dams exposed to polycyclic aromatic hydrocarbons (PAHs), reporting that increased PAH exposure was associated with increased weight, fat mass, higher gene expression of *PPARγ* and lower *PPARγ* CpG methylation [64]. In contrast to this inverse relationship between *PPARγ* methylation and weight, Drogan et al. (2015) analyzed subcutaneous adipose tissue (SAT) samples, showing that tissues from individuals with higher visceral fat mass had increased *PPARγ* CpG methylation [63]. Additionally, Nilsson et al. (2014) found differential *PPARγ* methylation in adipose tissues from subjects with type 2 diabetes compared to controls but did not report on this relationship’s direction [65]. We did not find that *PPARγ* methylation at birth could predict 9-year BMI z-score and more work is needed to further elucidate its role in *PPARγ* function and adipogenesis over time. Of note, site 1 was located in the north shore, further emphasizing the potentially critical role of north shore sites in regulating gene expression.

Lastly, there are several important points to consider with respect to our analyses. We measured methylation in blood samples, which can introduce bias if cell heterogeneity affects both methylation and obesity. However, our sensitivity analyses accounting for differences in cell composition did not substantially alter associations between *PPARγ* methylation and child size. Furthermore, our data displayed a consistent pattern of CpG methylation in blood samples over both birth and 9-year time points suggesting that heterogeneity of blood cell types may not significantly affect *PPARγ* methylation.

Nevertheless, whether *PPARγ* methylation in blood is a suitable marker for its activity in adipocytes remains unknown. Several studies have indicated that molecular changes in blood do reflect pathological changes in the body and gene expression in blood is highly concordant (>80%) with expression in other tissues [66, 67, 68–70]. With respect to body size, Ghosh et al. (2010) used principal components analysis to show that blood-based gene expression signals could distinguish between obese and lean subjects [71]. Interestingly, Charriere et al. (2003) found that based on transcriptome profiling, pre-adipocytes were more closely related to macrophages than adipocytes [72]. Further, a large genome-wide association study found that BMI was associated with methylation of *HIF3A* in both blood and adipose tissue [70]. Taken together, these data suggest that assessing *PPARγ* function in blood may be biological relevant however more work is needed to determine this in the context of methylation.

Additionally, although we had previously shown that methylation at *PPARγ* site 23 (gene body) was associated with *PPARγ* gene expression [27], this site was not significantly associated with child birth weight or BMI. Reasons for this inconsistency remain unclear and further research is warranted to examine relationships between CpG location and potential effects on gene expression. Overall, while our research argues that *PPARγ* methylation has a relationship with child body weight and that north shore sites may be of particular functional importance, key questions remain on factors that influence site-specific methylation and whether it can be used to predict metabolic outcomes over time.

## Conclusion

In summary, *PPARγ* CpG methylation is highly organized and conserved over time. We found high inter-CpG correlations between sites 1–3 (methylation block 1) and also between sites 18–23 (methylation block 2) for both birth and 9-year time points. Additionally, we report that methylation at birth for sites 1 (north shore) and 20 (gene body) was significantly and inversely associated with birth weight. Similarly, we found that methylation at these sites at 9 years was also significantly and inversely related to 9-year BMI z-score. Taken together, these results indicate that *PPARγ* methylation may be associated with child body size.

## Additional files

Additional file 1: Figure S1.
*PPARγ* Promoter and Gene Body CpG Sites. **Figure S1** shows the distribution of 23 CpG sites (red squares) measured by the Illumina Methylation 450 K Array. Blue squares indicate all other CpG sites (*N* = 183). Numbers in the DNA sequence, such as “2100” are shorthand for number of base pairs. (PPTX 295 kb)
Additional file 2: Table S1. Associations between perinatal characteristics and *PPARγ* methylation at birth. (PNG 42 kb)
Additional file 3: Table S2. Associations between perinatal characteristics and *PPARγ* methylation at 9 years. (PNG 50 kb)

## Abbreviations

BMI: Body mass index
CDC: Centers for disease control and prevention
CHAMACOS: Center for the Health Assessment of Mothers and Children of Salinas
CpG: Cytosine-guanine dinucleotides
CV: Coefficient of variation
DAGs: Directed acyclic graphs
DNA: Deoxyribonucleic acid
EWAS: Epigenome-wide association study
GEE: Generalized estimating equations
HIF3A: Hypoxia-inducible factor 3-alpha
IAP: Intracisternal A particle
MXL: Mexican ancestry in Los Angeles, California
MZ: Monozygotic
PAHs: Polycyclic aromatic hydrocarbons
PON1: Paraoxonase 1 gene
PPARγ: Peroxisome proliferator-activated receptor γ
SAT: Subcutaneous adipose tissue
SNP: Single nucleotide polymorphism
UTR: Untranslated region
