# Supplementary material for: CpG Methylation across the adipogenic PPARγ gene and its relationship with birthweight and child BMI at 9 years
Source: BMC Med Genet. 2017 Jan 26;18:7. doi: 10.1186/s12881-016-0365-4 (PMC5267417; doi:10.1186/s12881-016-0365-4)
Supplement: Additional file 1: Figure S1. — PPARγ Promoter and Gene Body CpG Sites. Figure S1 shows the distribution of 23 CpG sites (red squares) measured by the Illumina Methylation 450 K Array. Blue squares indicate all other CpG sites (N = 183). Numbers in the DNA sequence, such as “2100” are shorthand for number of base pairs. (PPTX 295 kb) [file 12881_2016_365_MOESM1_ESM.pptx]

## Slide 1
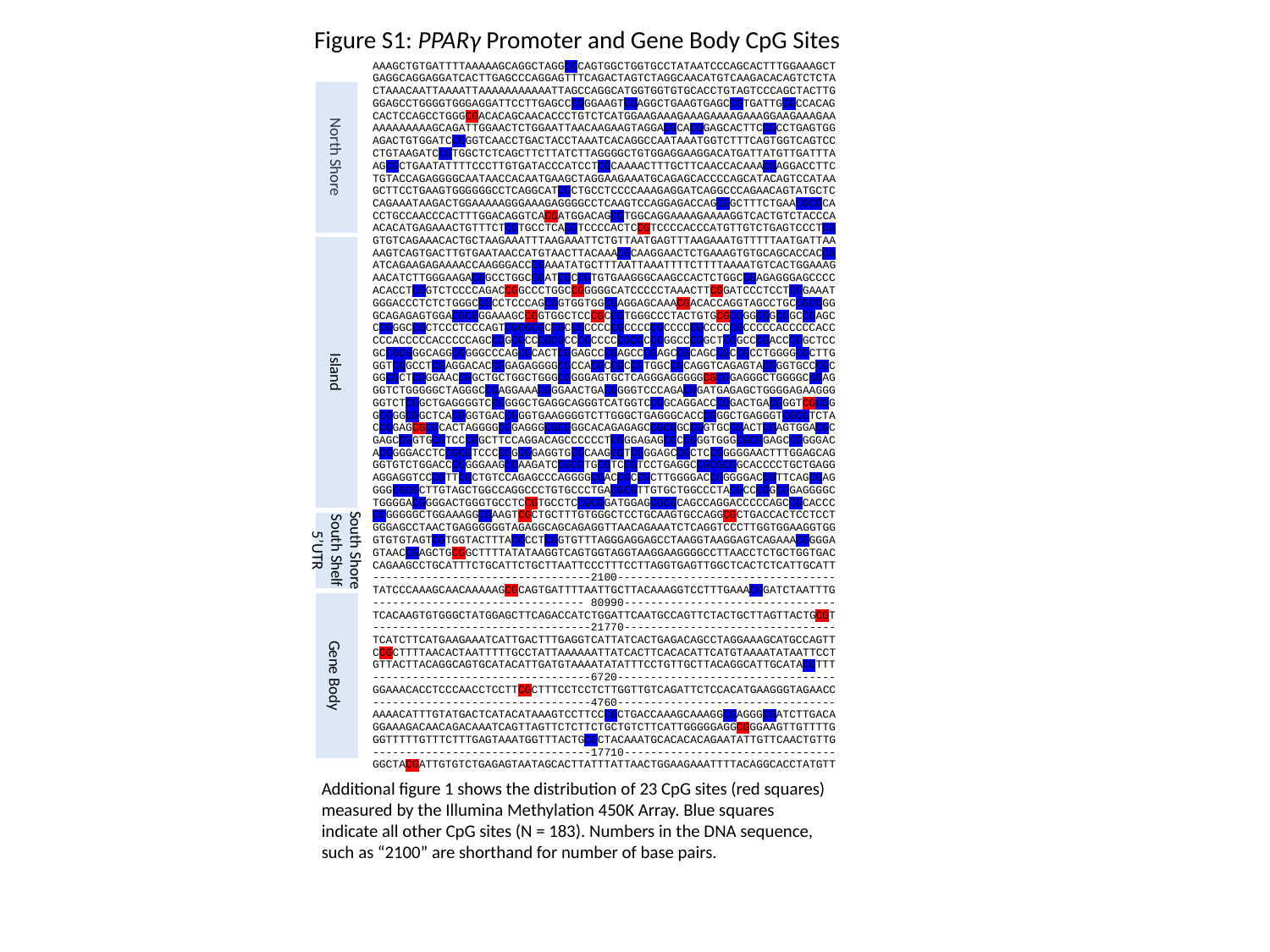

Figure S1: PPARγ Promoter and Gene Body CpG Sites
North Shore
Island
South Shore
South Shelf
5’UTR
Gene Body
Additional figure 1 shows the distribution of 23 CpG sites (red squares) measured by the Illumina Methylation 450K Array. Blue squares indicate all other CpG sites (N = 183). Numbers in the DNA sequence, such as “2100” are shorthand for number of base pairs.
