# Supplementary figures and images for: CpG Methylation across the adipogenic PPARγ gene and its relationship with birthweight and child BMI at 9 years
Source: BMC Med Genet. 2017 Jan 26;18:7. doi: 10.1186/s12881-016-0365-4 (PMC5267417; doi:10.1186/s12881-016-0365-4)

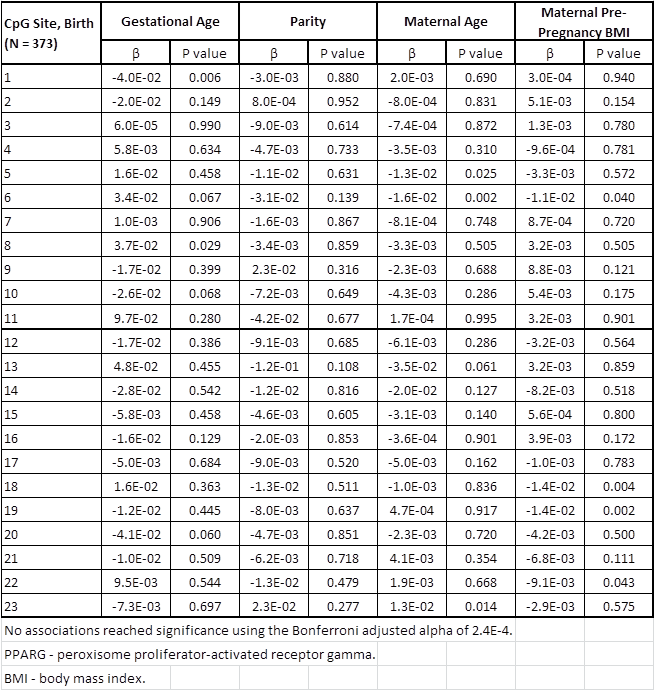

Supplement: Additional file 2: Table S1. — Associations between perinatal characteristics and PPARγ methylation at birth. (PNG 42 kb) [file 12881_2016_365_MOESM2_ESM.png]

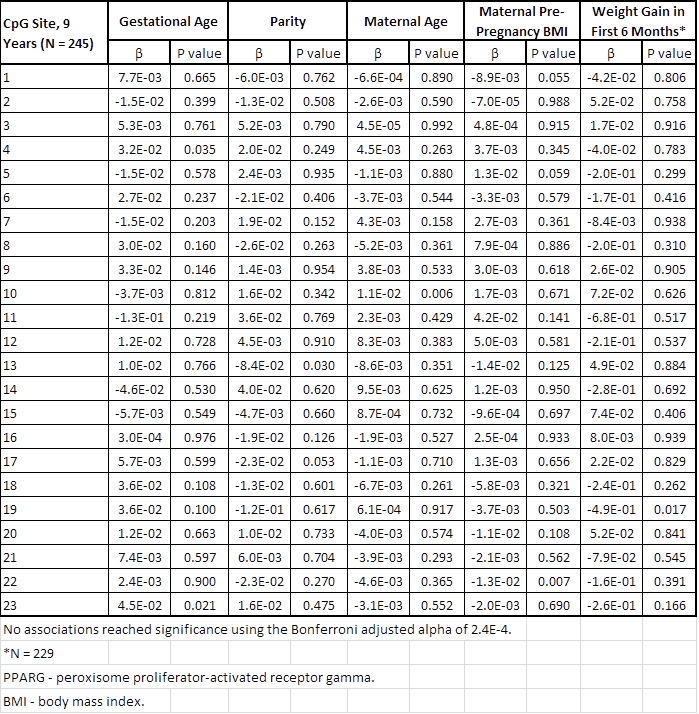

Supplement: Additional file 3: Table S2. — Associations between perinatal characteristics and PPARγ methylation at 9 years. (PNG 50 kb) [file 12881_2016_365_MOESM3_ESM.png]
